# Adipose-Derived Stem Cell Secretome Attenuates Eosinophilic Inflammation in a Chronic Rhinosinusitis with Nasal Polyps Mouse Model

**DOI:** 10.3390/ijms262412137

**Published:** 2025-12-17

**Authors:** Ji-Hwan Park, Hye-Jin Park, Dae Woo Kim, Sung-Dong Kim, Sue Jean Mun, Kyu-Sup Cho

**Affiliations:** 1 Department of Otorhinolaryngology and Research Institute for Convergence of Biomedical Science and Technology, Pusan National University Yangsan Hospital, Yangsan 50612, Republic of Korea; nobleivy@naver.com (J.-H.P.); baskie23@naver.com (S.J.M.); 2 Department of Otorhinolaryngology and Biomedical Research Institute, Pusan National University School of Medicine, Pusan National University Hospital, 179 Gudeok-Ro, Seo-gu, Busan 49241, Republic of Korea; kamdy11v@naver.com (H.-J.P.); applekims@hanmail.net (S.-D.K.); 3 Department of Otorhinolaryngology, Seoul Metropolitan Government Seoul National University Boramae Medical Center, Seoul 07061, Republic of Korea; kicubi73@snu.ac.kr

**Keywords:** immunosuppression, secretome, mesenchymal stem cells, nasal polyps, rhinosinusitis, mice, eosinophils, chemokine CCL11, cytokines

## Abstract

Adipose-derived stem cells (ASCs) and their secretome have been reported to improve allergic airway inflammation. Eosinophilic chronic rhinosinusitis with nasal polyps (ECRSwNP) is characterized by type 2 helper T (Th2)-diven inflammation, which shares similar mechanisms with allergic airway diseases. We assessed the immunomodulatory effects of ASC secretome on an ECRSwNP mouse model. ECRSwNP was induced by ovalbumin (OVA) and *Staphylococcus aureus* enterotoxin B (SEB) intranasal challenges in five-week-old BALB/c mice. To evaluate the effect of ASC secretome on eosinophilic nasal inflammation, 10 μg/50 μL of ASC-conditioned media were administered three times a week during the eight weeks. H&E and Sirius red staining were performed to evaluate the formation of nasal polyps (NPs) and the infiltration of eosinophils. The cytokine levels of interleukin (IL)-4, IL-5, IL-13, interferon-γ, IL-8, and eotaxin-1 were measured using ELISA(eBiosciences, San Diego, CA, USA). The expression levels of IL-8 and eotaxin-1 mRNA were determined by quantitative PCR. Eosinophil cationic protein (ECP) and eotaxin-1 expression were assessed by immunohistochemistry. Intranasal administration of ASC secretome significantly decreased NP-like formation and eosinophilic infiltration in the sinonasal mucosa of ECRSwNP mice. The increased IL-4, IL-5, and eotaxin-1 levels after OVA + SEB challenge remarkably decreased by ASC secretome treatment. Furthermore, ASC secretome notably decreased the gene expression of eotaxin-1 by PCR, as well as ECP and eotaxin-1 expression by immunohistochemistry. ASC secretome had immunomodulatory effects in a mouse model of ECRSwNP. Intranasal administration of ASC secretome resulted in a significant reduction in NP formation and eosinophilic inflammation through the suppression of IL-4, IL-5, eotaxin-1, and ECP.

## 1. Introduction

Chronic rhinosinusitis (CRS) is an inflammatory condition affecting the lining of nasal cavities and paranasal sinuses with various signs and symptoms such as nasal obstruction, rhinorrhea, or decreased sense of smell lasting more than 12 weeks [1]. CRS is one of the most common chronic diseases across the world, with an estimated prevalence of 5–15% affecting all age groups [1]. CRS remains a significant public health problem related to quality of life with a considerable socioeconomic burden [2]. In the phenotypic classification according to the presence of nasal polyps (NPs), patients with CRS with nasal polyps (CRSwNP) had a greater burden of symptoms, more prior surgery, and greater use of medications compared with CRS without nasal polyps (CRSsNP) [3].

CRS can be classified into three endotypes based on the presence of type 1, type 2, and type 3 inflammation [4]. Type 2 endotype is driven by activation of Th2 inflammatory pathway producing cytokines such as interleukin (IL)-4, IL-5, and IL-13, whereas type 1 and type 3 involve the activation of Th1 and Th17 pathways associated with increased levels of interferon (IFN)-γ and IL-17, respectively [5]. Eosinophilic CRS (ECRS) is a type 2 CRS characterized by Th2 immune response and severe eosinophilic infiltration in the sinonasal mucosa [6]. ECRS often presents with multiple and bilateral NPs in comparison to non-ECRS [7]. Non-ECRSwNP can be relatively well managed with endoscopic sinus surgery (ESS) combined with low-dose macrolide therapy [8]. However, ECRSwNP has been refractory to the maximal medical treatment including intranasal or systemic corticosteroids and demonstrated a high propensity to recur after ESS [8]. Recently, biological agents including omalizumab, dupilumab, or mepolizumab are being considered as new treatment options for type 2 inflammation in ECRSwNP [9].

Mesenchymal stem cells (MSCs) including those derived from adipose tissue have the ability to suppress inflammation and immune response [10]. Several studies have shown that adipose-derived stem cells (ASCs) and other MSCs ameliorated allergic airway inflammation in the allergic rhinitis and bronchial asthma mouse model [11,12]. Moreover, the secretome released from ASCs and their extracellular vesicles (EVs) have been shown to exert comparable therapeutic effects to ASCs in the attenuation of allergic airway inflammation [13,14]. ECRSwNP is categorized as a type 2 inflammatory disease due to its similarity in the pathogenesis of allergic rhinitis and asthma, and has often been associated with them [15]. However, no studies have evaluated the immunomodulatory effects of secretome acquired from ASCs on Th2-mediated ECRSwNP.

We established an ECRSwNP mouse model based on ovalbumin (OVA) + *Staphylococcus aureus* enterotoxin B (SEB) intranasal challenges and assessed the immunomodulatory effects of intranasally administered ASC secretome on eosinophilic inflammation, cytokine levels, and chemokine gene expression in an ECRSwNP murine model.

## 2. Results

### 2.1. NP-like Lesion Formation

**Figure 1:**
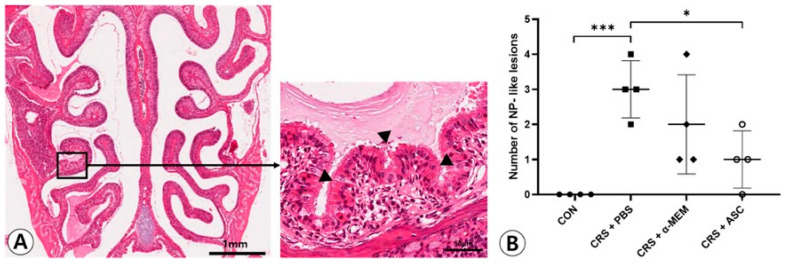
Effect of adipose-derived stem cells (ASC) secretome on the nasal polyp (NP) formation. (**A**) Mucosal bulges with eosinophilic infiltration (arrowheads) were considered as NP-like lesions (hematoxylin and eosin, ×20, ×400). (**B**) The number of NP-like lesions decreased significantly in the CRS + ASC group compared to CRS + PBS group. Data are expressed as the means ± standard error of the mean. Only statistically significant differences are indicated. CON, control; CRS, chronic rhinosinusitis; MEM, modified Eagle’s medium; NP, nasal polyp; PBS, phosphate-buffered saline, * *p* < 0.05, *** *p* < 0.001.

NP-like lesions were mostly found at the transition zone between the olfactory and respiratory epithelium (Figure 1A). There were no NP-like lesions in the CON group. In total, 12 and eight lesions were observed in four CRSwNP mice treated with PBS or α-MEM, respectively. The NP-like lesions were significantly higher in number in the CRS + PBS group compared to the CON group (*p* < 0.001). However, intranasal treatment of ASC secretome markedly lowered the numbers of NP-like lesions in the CRSwNP (*p* = 0.013) (Figure 1B).

### 2.2. Eosinophilic Inflammation in Sinonasal Mucosa

**Figure 2:**
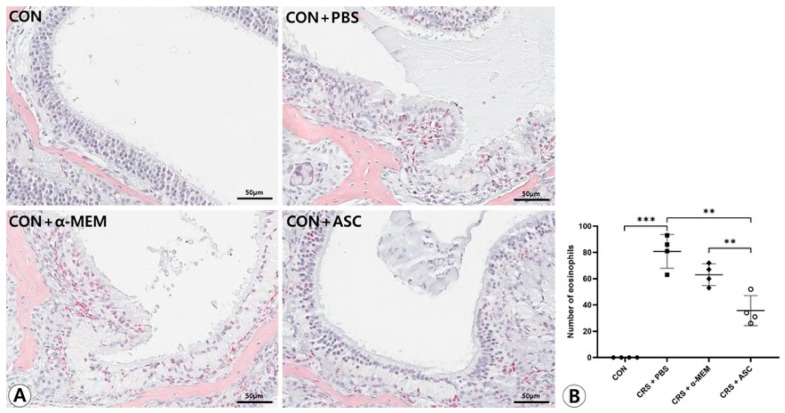
Effect of adipose-derived stem cell (ASC) secretome on eosinophilic nasal inflammation. (**A**) Mucosal thickening and eosinophilic infiltration were observed in the CRS + PBS, CRS + α-MEM, and CRS + ASC groups (Sirius red, ×400). (**B**) Eosinophilic infiltration decreased significantly in the CRS + ASC group compared with those in the CRS + PBS and CRS + α-MEM groups. Data are expressed as means ± standard error of the mean. Only statistically significant differences are indicated. CON, control; CRS, chronic rhinosinusitis; MEM, modified Eagle’s medium; PBS, phosphate-buffered saline. ** *p* < 0.01, *** *p* < 0.001.

There was no infiltration of eosinophils in the sinonasal mucosa in the CON group. Eosinophilic inflammation and mucosal thickening were significantly increased in the CRSwNP mice. However, no obvious infiltration of eosinophils was found in the CRSwNP mice treated with ASC secretome (Figure 2A). The number of eosinophils was markedly decreased in the CRS + ASC group compared to the CRS + PBS and CRS + α-MEM groups (*p* = 0.002 and *p* = 0.008, respectively) (Figure 2B).

### 2.3. Analysis of Cytokines Expression

**Figure 3:**
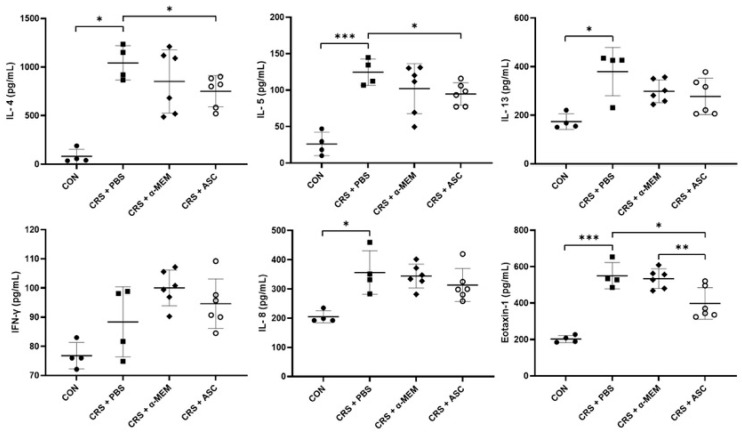
Effect of adipose-derived stem cell (ASC) secretome on cytokine levels in the sinonasal mucosa. IL-4, IL-5, IL-13, IL-8, and eotaxin-1 levels were significantly increased in the CRS + PBS group compared to the CON group. Intranasal administration of ASC secretome markedly reduced the levels of IL-4, IL-5, and eotaxin-1. Data are expressed as means ± standard error of the mean. Only statistically significant differences are indicated. CON, control; CRS, chronic rhinosinusitis; IFN, interferon; IL, interleukin; MEM, modified Eagle’s medium; PBS, phosphate-buffered saline. * *p* < 0.05, ** *p* < 0.01, *** *p* < 0.001.

The expression levels of cytokines (IL-4, IL-5, IL-13, and IFN-γ) and chemokines (IL-8 and eotaxin-1) were evaluated with ELISA. The levels of IL-4, IL-5, IL-13, IL-8, and eotaxin-1 were markedly elevated in the CRS + PBS group compared to the CON group (*p* = 0.029, *p* < 0.001, *p* = 0.029, *p* = 0.029, and *p* < 0.001, respectively). However, intranasal ASC secretome treatment significantly lowered the IL-4 and IL-5 levels in the sinonasal mucosa of the CRS + PBS group (*p* = 0.027 and *p* = 0.023, respectively). Furthermore, the expression of eotaxin-1 was significantly lower in the CRS + ASC group than in the CRS + PBS and CRS + α-MEM groups (*p* = 0.021 and *p* = 0.009, respectively) (Figure 3).

### 2.4. Analysis of Chemokine Gene Expression

**Figure 4:**
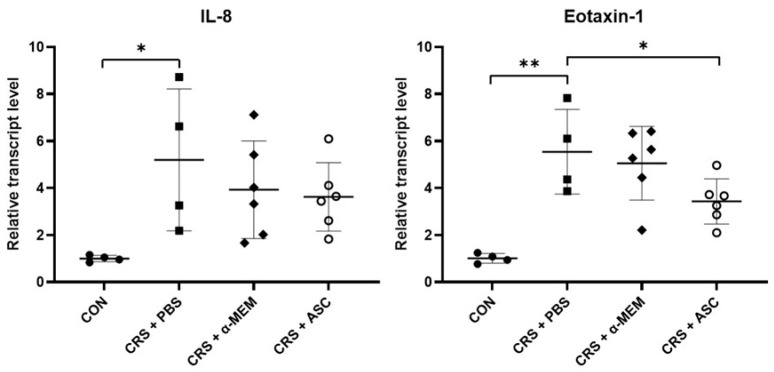
Effect of adipose-derived stem cells (ASC) secretome on chemokine gene expression in the sinonasal mucosa. The mRNA expression levels of IL-8 and eotaxin-1 were significantly increased in the CRS + PBS group compared to the CON group. Treatment of ASC secretome remarkably reduced eotaxin-1 expression in the CRS + ASC group compared with the CRS + PBS group. Data are expressed as means ± standard error of the mean. Only statistically significant differences are indicated. CON, control; CRS, chronic rhinosinusitis; IL, interleukin; MEM, modified Eagle’s medium; PBS, phosphate-buffered saline. * *p* < 0.05, ** *p* < 0.01.

The mRNA expression levels of chemokines including IL-8 and eotaxin-1 were evaluated with RT-qPCR. The gene expressions of IL-8 and eotaxin-1 were remarkably increased in the CRS + PBS group compared to the CON group (*p* = 0.032 and *p* = 0.002, respectively). However, intranasal administration of ASC secretome significantly decreased eotaxin-1 expression in the CRSwNP (*p* = 0.041) (Figure 4).

### 2.5. Expression of ECP and Eotaxin-1

**Figure 5:**
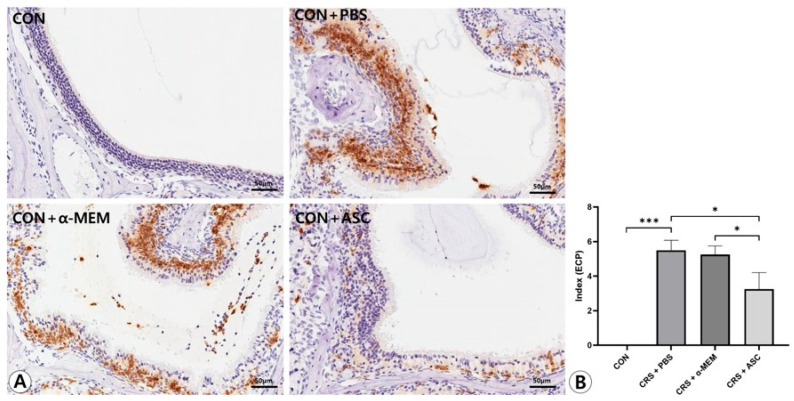
Effect of adipose-derived stem cell (ASC) secretome on eosinophil cationic protein (ECP) expression in the sinonasal mucosa. (**A**) The ECP expression was more diffuse and clearly stronger in the CRS + PBS group than in the CON group, but this was significantly decreased by the intranasal administration of ASC secretome (immunohistochemistry, ×400). (**B**) In semi-quantitative comparative analysis, intranasal administration of ASC secretome markedly decreased ECP expression in the CRSwNP mice. Only statistically significant differences are indicated. CON, control; CRS, chronic rhinosinusitis; MEM, modified Eagle’s medium; PBS, phosphate-buffered saline. * *p* < 0.05, *** *p* < 0.001.

**Figure 6:**
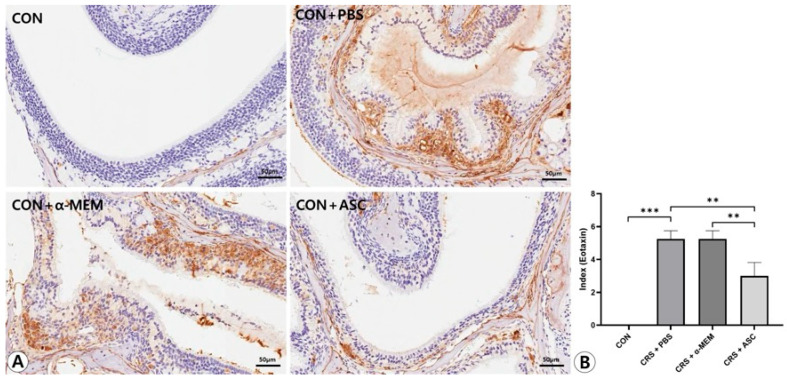
Effect of adipose-derived stem cells (ASC) secretome on eotaxin-1 expression in the sinonasal mucosa. (**A**) The eotaxin-1 expression was more diffuse and clearly stronger in the CRS+ PBS group compared to the CON group, but this was significantly decreased by the intranasal administration of ASC secretome (immunohistochemistry, ×400). (**B**) In semi-quantitative comparative analysis, intranasal administration of ASC secretome markedly decreased eotaxin-1 expression in the CRSwNP mice. Only statistically significant differences are indicated. CON, control; CRS, chronic rhinosinusitis; MEM, modified Eagle’s medium; PBS, phosphate-buffered saline. ** *p* < 0.01, *** *p* < 0.001.

The expression levels of ECP and eotaxin-1 in the sinonasal mucosa were assessed by immunohistochemical staining. ECP and eotaxin-1 expression were more diffuse and clearly stronger in the CRS + PBS group compared to the CON group, but were markedly decreased following intranasal administration of ASC secretome. In the semi-quantitative analysis, the expression levels of ECP and eotaxin-1 were significantly increased in the CRS + PBS group compared to the CON group (*p* < 0.001). However, intranasal ASC secretome treatment remarkably decreased ECP and eotaxin-1 expression in the CRSwNP mice (*p* = 0.010 and *p* = 0.003, respectively) (Figure 5 and Figure 6).

## 3. Discussion

The MSC secretome is the totality of all messenger substances released by MSC to the outside, including the soluble factors and EVs [16]. The MSC secretome contains a variety of components, such as cytokines, chemokines, growth factors, and extracellular matrix proteins that provide a similar therapeutic effect as systemically administered MSCs [13,17]. MSC secretome has been reported as a promising candidate to treat allergic airway diseases as it can modulate immune function [16,18]. ASC secretome or ASC-derived EVs have improved allergic airway inflammation by suppressing the production of Th2 cytokines and inducing the expansion of regulatory T cells (Treg) [13,14]. Furthermore, compared with ASC treatment, cell-free therapy mediated by ASC secretome has many advantages including safety, ease of handling or storage, lower possibility of immune rejection, and no risk of vascular occlusion [19].

ECRS and non-ECRS display distinct features on the basis of histopathology and the expression of inflammatory mediators. ECRS has been characterized by Th2-skewed eosinophilic inflammation with high levels of IL-5 and defective functions of Treg, whereas non-ECRS showed a predominant Th1 milieu with high IFN-γ and adequate Treg function [20]. An NP is a chronic inflammatory disease characterized by an edematous mass of hyperplastic epithelium and lamina propria prolapsing into the nose. It is clinically challenging to manage the NP due to its tendency to recur frequently [21,22]. Based on the immunosuppressive effects of ASC secretome on Th2-mediated eosinophilic inflammation through inhibition of Th2 cell activity in allergic rhinitis and asthma, we hypothesized that ASC secretome would be capable of regulating eosinophilic inflammation and the production of related cytokines in ECRSwNP. To our knowledge, this is the first study to investigate the immunomodulatory effects of ASC secretome in an ECRSwNP mouse model.

In this study, the ECRSwNP mouse model was established by OVA + SEB intranasal challenges using BALB/c mice. OVA-challenged mice were exposed to SEB for NP formation during the last 8 weeks. NP-like structures were present mainly at the junction of olfactory and respiratory epithelium. NP-like lesions were increased in the mice treated with OVA + SEB compared to the mice treated with PBS. Eosinophilic inflammation and mucosal thickening were significantly increased in the CRSwNP mice. However, intranasal administration of ASC secretome significantly reduced the NP formation and eosinophilic infiltration in the sinonasal mucosa of CRSwNP.

Intranasal OVA + SEB challenges have been reported to induce Th2-mediated eosinophilic reaction through the activation of cytokines and chemokines [23,24]. The levels of IL-4, IL-5, IL-13, IL-8, and eotaxin-1 were markedly increased in this ECRSwNP mouse model. IL-4, IL-13, and IL-5 are central Th2 cytokines with distinct roles in the pathogenesis of CRSwNP [25]. Of these cytokines, IL-5, together with eotaxins, plays a critical role in recruiting eosinophils to the bloodstream [26]. Specifically, eotaxin-1 (CCL11), eotaxin-2 (CCL24), and eotaxin-3 (CCL26) are key chemokine molecules that trigger migration of eosinophils to inflammatory sites, such as the lung and the sinonasal mucosa [26,27]. IL-8 is a powerful chemoattractant for both neutrophils and eosinophils in CRS [28]. Not only non-ECRS but also ECRS accompanying severe inflammation is reported to show neutrophilic inflammation [4]. Taken together with the NP-like lesions and eosinophilic inflammation by OVA + SEB instillation, these findings indicate that the severe ECRSwNP model was successfully induced.

The present study demonstrated that ASC secretome delivered through the intranasal route significantly decreased the IL-4, IL-5, and eotaxin-1 levels in the sinonasal mucosa of ECRSwNP. Furthermore, eotaxin-1 gene expression by RT-qPCR, as well as ECP and eotaxin-1 expression by immunohistochemistry, were markedly reduced after ASC secretome treatment. IL-13, IL-8, and IFN-γ also increased during disease induction; however, these cytokines are not central mediators of the eosinophilic Th2 response. IL-13 may reflect broader remodeling-related pathways, IL-8 is more closely associated with neutrophil-driven inflammation, and IFN-γ represents Th1 activity with limited involvement in this Th2-skewed context. These characteristics might explain why ASC secretome treatment, despite clearly reducing IL-4, IL-5, and eotaxin-1, did not result in significant changes in IL-13, IL-8, or IFN-γ.

Importantly, the CRS and CRS + α-MEM groups showed no differences across all evaluated inflammatory markers, indicating that α-MEM itself had no effect and confirming that the anti-inflammatory changes were attributable to the ASC secretome. These results are consistent with the immunomodulatory effects of ASCs on T lymphocytes and cytokine expression in eosinophilic NPs [29]. ASC treatment significantly decreased the activated T lymphocytes and Th2 cytokines in co-cultured infiltrating cells from NPs with ASC in vitro [30]. Together with previous in vitro findings, our results provide evidence that ASC secretome improved eosinophilic inflammation by downregulation of IL-4, IL-5, and eotaxin-1 in the ECRSwNP murine model.

Recent studies of mesenchymal stromal cell-derived EVs provide plausible explanations for how ASC secretome may modulate Th2-driven eosinophilic inflammation. In allergic airway models, EVs derived from human MSCs have been shown to reduce eosinophil-dominant inflammation by reshaping innate immune responses, particularly through the modulation of pulmonary macrophage polarization and the attenuation of type 2 cytokine signaling [30]. These macrophage-mediated regulatory effects can diminish eosinophil recruitment and activation, which is consistent with the decreases in eotaxin-1 and ECP observed in our ECRSwNP model.

In addition, EVs from hypoxia-conditioned MSCs have been reported to suppress IL-4 and IL-13-driven airway inflammation through the delivery of anti-inflammatory molecular cargo, including miR-146a-5p [31]. A recent immunological review further highlights that MSC- and MSC-EV-derived factors can influence dendritic cell activation, T cell responses, and innate lymphoid cell-associated type 2 inflammation [32]. Taken together, these findings suggest that ASC secretome may regulate multiple immunological processes that converge to limit Th2-mediated eosinophilic inflammation, providing a biologically plausible basis for the reductions in IL-4, IL-5, eotaxin-1, and ECP observed in this study.

According to the modernized classification, chronic airway diseases such as ECRSwNP are now interpreted within a broader immunologic framework that incorporates not only inflammatory endotypes but also the underlying hypersensitivity mechanisms that initiate immune dysregulation [33]. In this framework, many eosinophil- and T cell-driven upper airway disorders are now considered non-IgE-mediated type IV hypersensitivity reactions, in which cytokine-mediated, cell-driven immune responses predominate even in the absence of IgE sensitization. The ECRSwNP murine model used in the present study reflects this type IV-like hypersensitivity environment, characterized by strong Th2 signaling and eosinophilic tissue inflammation arising from upstream cellular and cytokine interactions. Our findings suggest that the ASC secretome, which significantly reduced eosinophil infiltration and key type 2 cytokines, may modulate not only the downstream inflammatory cascade but also earlier immunologic processes associated with non-IgE–mediated hypersensitivity.

This study has several limitations. We were unable to distinguish which components of the ASC secretome contributed to the regulation of immune responses. Therefore, future research is necessary to identify the specific secretome-derived factors responsible for suppressing eosinophilic inflammation in ECRSwNP mice. Because ASC secretome contains a heterogeneous mixture of bioactive molecules, more detailed component-level characterization will be important in future studies and may also help improve reproducibility across investigations. In particular, mechanistic studies examining how individual ASC secretome components interact with T cell-mediated cytokine networks will be essential to clarify their potential roles in modulating hypersensitivity responses. Molecular and genetic approaches may further elucidate the immunomodulatory processes mediated by ASC secretome. Additionally, the plausible therapeutic role of ASC secretome in patients with ECRSwNP should be evaluated in future clinical studies to strengthen the translational relevance of our findings.

## 4. Materials and Methods

### 4.1. Animals

Four-week-old male BALB/c mice were purchased from HANA (Busan, Korea) and bred in a specific pathogen-free animal facility. After a week of stabilization, 5-week-old mice were divided into the control (CON) group and three experimental groups. The mice were monitored once daily by a veterinarian. Humane endpoints included ≥20% weight loss, severe lethargy, inability to eat or drink, or respiratory distress. No mice reached these endpoints. The animal study protocol was approved by the Institutional Animal Care and Use Committee of the Pusan National University Hospital (Approval No. PNUH-2023-218).

### 4.2. Isolation and Culture of ASCs

Adipose tissue was harvested from the inguinal region of eight-week-old male BALB/c mice, following previously described protocols [13,14]. The tissue was washed with phosphate-buffered saline (PBS) and digested with 0.075% collagenase type I (Sigma-Aldrich, St. Louis, MO, USA) at 37 °C for 30 min. The enzymatic reaction was neutralized by adding α-modified Eagle’s medium (α-MEM) containing 10% fetal bovine serum (FBS). The resulting suspension was centrifuged at 1200× *g* for 10 min to obtain a pellet, which was then incubated overnight at 37 °C in 5% CO_2_ using control medium composed of α-MEM, 10% FBS, 100 U/mL penicillin, and 100 μg/mL streptomycin. After incubation, non-adherent cells were removed by washing thoroughly with PBS. ASCs at the third or fourth passages were selected and used for subsequent analyses after phenotypic confirmation by flow cytometry, as described in earlier studies [34].

### 4.3. Collection of Conditioned Media Containing ASC Secretome

ASC supernatant was collected according to the previously reported protocols [13]. ASCs, at a concentration of 1 × 10^5^ cells/cm^2^, were cultured for about 48 h until they reached 1 × 10^6^ cells/cm^2^ in α-MEM containing 10% FBS at 37 °C in 5% CO_2_. The culture medium obtained from ASCs was centrifuged at 12,000× *g* for 30 min. The resulting supernatant, hereafter designated as the “ASC secretome”, was collected and subsequently lyophilized. To eliminate residual salts, the sample was further purified using HiTrap^®^ Desalting Columns (Cytiva, Uppsala, Sweden). Lipopolysaccharides were depleted (endotoxin levels < 0.01 μg/mL) from the ASC secretome by Detoxi-Gel™ Endotoxin Removing Columns (Thermo Fisher Scientific, Waltham, MA, USA) following the manufacturer’s instructions. The final concentration of protein in the ASC secretome was determined to be 0.2 mg/mL using a Bradford assay kit (Bio-Rad, Hercules, CA, USA).

### 4.4. Mouse Model of ECRSwNP and Intranasal Treatment of ASC Secretome

**Figure 7:**
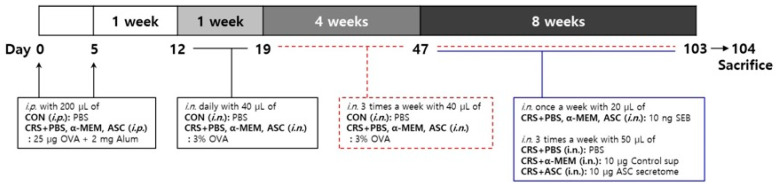
The experimental protocol. A mouse model of eosinophilic nasal inflammation was induced by prolonged intranasal instillation of ovalbumin (OVA), followed by weekly challenges with *Staphylococcus aureus* enterotoxin B (SEB). The treatment was administered intranasally three times weekly during the last eight weeks. The CRS + PBS, CRS + α-MEM, and CRS + ASC groups were treated with PBS, α-MEM, and ASC secretome, respectively. ASCs, adipose stem cells; CON, control; i.n., intranasal instillation; i.p., intraperitoneal injection; MEM, modified Eagle’s medium; PBS, phosphate-buffered saline.

Eosinophilic inflammation was induced in the sinonasal mucosa of a murine model according to the following steps as described in a previous study (Figure 7) [35]. On days 0 and 5, mice in the control (CON) group were given an intraperitoneal injection of 200 µL of PBS and mice in CRS + PBS, CRS + α-MEM, and CRS + ASC groups were sensitized by intraperitoneal injection with 25 µg of OVA (grade V; Sigma-Aldrich, St. Louis, MO, USA) and 2 mg of aluminum hydroxide (InvivoGen, San Diego, CA, USA) in a total volume of 200 µL. The CRS + α-MEM group was included as a vehicle control to exclude any potential effects of the culture medium itself, as α-MEM was used as the solvent for preparing the ASC secretome. One week after sensitization, on day 12, mice in the CON group were challenged intranasally with 40 µL of PBS and mice in CRS + PBS, CRS + α-MEM, and CRS + ASC groups with 40 µL of 3% OVA daily for a week and then three times a week for 12 consecutive weeks until day 103. During the last 8 weeks, mice in CRS + PBS, CRS + α-MEM, and CRS + ASC groups were given 10 ng SEB diluted in 20 μL PBS intranasally once a week after OVA instillation. Following administration of SEB, treatments were given to the experimental groups three times a week. The CRS + PBS group was treated intranasally with 50 μL PBS, the CRS + α-MEM group with fresh control medium supernatant (10 μg/50 μL), and the CRS + ASC group with ASC secretome (10 μg/50 μL).

Twenty-four hours after the final intranasal treatments, all mice were sacrificed using gradually filled CO_2_ chamber for analyses. From each group, four mice were selected for histologic evaluation. The sinonasal mucosa from the four mice of the CON and CRS + PBS groups each and the six mice of the CRS + α-MEM and CRS + ASC groups each was collected for analyses of cytokine and chemokine expression using enzyme-linked immunosorbent assays (ELISA, eBiosciences, San Diego, CA, USA) and reverse transcription-quantitative polymerase chain reaction (RT-qPCR).

### 4.5. Histopathologic Analysis of Sinonasal Mucosa

Mice were sacrificed by carbon dioxide inhalation, and the heads were dissected to remove the skin, soft tissues, and mandible. The obtained skulls were immersed in 4% paraformaldehyde at 4 °C for 72 h to allow fixation. Afterward, decalcification was carried out using 10% ethylenediaminetetraacetic acid (pH 7.4) for approximately two weeks. The tissues were then dehydrated sequentially through graded alcohols and xylene and subsequently embedded in paraffin. Coronal sections 4 μm thick were prepared from the paraffin blocks. For histological evaluation, sections were selected from the region between the incisive papilla and the second molar teeth, corresponding to the anatomical level where the ethmoid turbinates were most clearly developed. The maxillary sinus proper and the ethmoidal labyrinth were identified in sections located posterior to the second maxillary turbinelle. Two consecutive coronal sections with similar sinus cavities were selected for analyses. The formation of NP-like lesion and infiltration of eosinophils were evaluated by hematoxylin and eosin (H&E) and Sirius red staining, respectively. The criteria of NPs were based on the previous studies that included an edematous projection from the lining of the epithelium to the lumen with infiltration of eosinophils and microcavity formation [36,37]. Simple protruding lesions without infiltration of eosinophils were not considered to be NP-like lesions.

For the Sirius red staining, we followed the modification of the original method described by Llewellyn and eliminated the sodium chloride step. Briefly, the sections were placed in Harris hematoxylin solution for nuclear staining. These were rinsed with tap water, and then with 100% ethanol. The sections were immersed in an alkaline Sirius red solution for 3 h and rinsed with tap water [38]. Two independent observers who were blinded to the group assignment counted the number of NP-like lesions and eosinophils in a high-power field (×400 magnification). Two consecutive slides were examined to prevent any processing errors.

### 4.6. Expression of Cytokines in the Sinonasal Mucosa

The extract was obtained from a portion of the dissected sinonasal mucosa using radioimmunoprecipitation assay lysis buffer (Sigma, St. Louis, MO, USA). The protein of the extract was quantitated by Bradford assay kit (Bio-Rad, Hercules, CA, USA) according to the manufacturer’s instructions. Equal amounts of each sample were used in the assay. The expression levels of IL-4, IL-5, IL-13, IFN-γ (eBioscience, San Diego, CA, USA), IL-8 (Assay Genie, Dublin, Ireland), and eotaxin-1 (R&D Systems, Minneapolis, MN, USA) in the extract of sinonasal mucosa were determined by ELISA kits according to the manufacturer’s recommendations (eBiosciences). The absorbance of the final reactant was measured at 450 nm using an ELISA plate reader.

### 4.7. Chemokine Gene Expression in the Sinonasal Mucosa

The transcript levels of chemokine CXCL8 (IL-8) and CCL11 (eotaxin-1) were determined by RT-qPCR. Total RNA from portions of the dissected sinonasal mucosa was extracted using QIAzol (Qiagen, Hilden, Germany) according to the protocols of the manufacturer. RT-qPCR was performed using an iCycler^™^ (Bio-Rad, Hercules, CA, USA) in a LightCycler 96 real-time PCR System (Roche, Basel, Switzerland). GAPDH was used as a reference gene. Table 1 shows the list of primers used for real-time PCR. The conditions for amplification were 95 °C for 1 min 30 s, denaturation at 95 °C for 25 s, primer annealing at 50–55 °C for 20 s, and elongation at 72 °C for 30 s for 40 cycles.

### 4.8. Immunohistochemistry

Eosinophil cationic protein (ECP) and eotaxin-1 expression were assessed by semi-quantitative analysis of immunohistochemistry. For immunohistochemical staining, sections were washed with PBS and blocked with 2% bovine serum albumin (Sigma, St. Louis, MO, USA)/PBS to remove nonspecific binding. The sections were incubated with purified anti-ECP immunoglobulin G (IgG) (Santa Cruz Biotechnology, Santa Cruz, CA, USA) and anti-CCL11 IgG (Santa Cruz Biotechnology) primary polyclonal antibody overnight at 4 °C. After incubation, sections were washed with PBS and then incubated with biotin-conjugated anti-rabbit IgG (Santa Cruz Biotechnology) for 1 h at room temperature. Sections with bound antibodies were detected with the peroxidase substrate kit (Vectastain ABC avidin/biotin Complex kit, Vector Laboratories, Burlingame, CA, USA). The sections were washed, counterstained with hematoxylin, and examined by light microscopy. Cytoplasmic staining was recorded by the semi-quantitative grading system considering both the intensity of the staining and the proportion of stained cells by two independent examiners blinded to the groups at a high-power field (×400 magnification). The intensity was recorded as 0 (no staining), 1 (light staining), 2 (moderate staining), or 3 (intense staining). The proportion of tissue sample with a positively stained cytoplasmic area was recorded as 0 (no cells were positive), 1 (positive staining in <10% of cells), 2 (positive staining in 10–50% of cells), or 3 (positive staining in >50% of cells). A staining index was calculated as the sum of the staining intensity and the proportion of stained cells [39].

### 4.9. Statistical Analysis

All data are presented as the mean ± standard error of the mean. Group comparisons were performed using either the Student’s *t*-test or the Mann–Whitney U test, as appropriate. Statistical analyses were carried out with SPSS software for Windows (ver. 28.0; SPSS Inc., Chicago, IL, USA), and graphical illustrations were produced using GraphPad Prism (ver. 10.2.3; GraphPad Software, San Diego, CA, USA). A *p*-value less than 0.05 was considered statistically significant.

## 5. Conclusions

This study showed the immunomodulatory effects of ASC secretome on eosinophilic sinonasal inflammation induced by OVA and SEB challenge in mice. Intranasally administered ASC secretome significantly decreased NP-like lesions and eosinophilic inflammation in an ECRSwNP mouse model through the suppression of IL-4, IL-5, and eotaxin-1 and ECP.

## Figures and Tables

**Table 1:** Primer sequences used for real-time PCR.

Primer	Sequence
GAPDH-for	5′-TAC CCC CAA TGT GTC CGT C-3′
GAPDH-rev	5′-AAG AGT GGG AGT TGC TGT TGA AG-3′
IL-8-for	5′-CTA GGC ATC TTC GTC CGT CC-3′
IL-8-rev	5′-TTC ACC CAT GGA GCA TCA GG-3′
Eotaxin-1-for	5′-GCG CTT CTA TTC CTG CTC ACG G-3′
Eotaxin-1-rev	5′-GTG GCA TCC TGG ACC CAC TTC TTC-3′

IL, interleukin; for, forward; rev, reverse.

## Data Availability

The original contributions presented in this study are included in the article. Further inquiries can be directed to the corresponding author.
